# Evaluation of effectiveness of a regional remote antimicrobial stewardship program

**DOI:** 10.1017/ash.2026.10383

**Published:** 2026-05-11

**Authors:** Taylor Grubish, David Schmidt, Sara Badaglialacqua, Seth Warner, Daniel Morris, Chris Destache, Rima El-Herte, Michael Koren

**Affiliations:** 1 Department of Pharmacy Services, CHI Health, A Member of CommonSpirit Health, USA; 2 Creighton University School of Medicine, USA; 3 Creighton University School of Pharmacy and Health Professions, USA

## Abstract

**Objective:**

Antimicrobial stewardship programs (ASPs) are designed to optimize antimicrobial use for healthcare facilities and are required at all acute care and critical access hospitals (CAHs). However, rural CAHs often lack the resources to implement robust ASPs. The purpose of this study was to evaluate the impact of adding regional remote antimicrobial stewardship services across a 14-hospital system consisting of one acute care and thirteen CAHs.

**Methods:**

Remote ASP expansion consisted of adding an infectious disease (ID) pharmacist and two ID physicians (0.3 FTE) to provide daily stewardship activities including prospective feedback on antibiotic optimization and use education through remote chart review and communication. Antimicrobial utilization, cost savings, and *Clostridioides difficile* infection (CDI) rates were tracked. Intervention data and acceptance rates were also collected.

**Results:**

In the first twelve months following regional remote stewardship expansion, the ASP demonstrated a $619,053.97 reduction (56% decrease in Antibiotic Cost/CMI adjusted patient days) in antimicrobial expenses. While only aztreonam use was significantly reduced at the acute care facility (*P* < .01), the use of vancomycin, meropenem, linezolid, and aztreonam decreased by 33%, 31%, 18%, and 35%, respectively, among the CAHs (*P* < .001). A 53% reduction in CDI rates was observed across the health system (*P* < .01). The program averaged 92 interventions per month, with an overall intervention acceptance rate of 86%.

**Conclusions:**

Remote ASPs represent a viable strategy for extending antibiotic stewardship expertise to resource-limited settings to achieve financial and clinical benefits.

## Introduction

Inappropriate antibiotic use remains a barrier to optimal patient care, with studies finding that between 20 and 50% of the antibiotics prescribed are either inappropriate or unnecessary.^1,2^ This misuse leads to increased costs of healthcare as well as contributes to the rising incidence of *Clostridioides difficile* infections (CDI) across the US, with annual infections now totaling more than 450,000, resulting to between 12,000–30,000 deaths, and costing US hospitals between $1.0–4.9 billion each year.^3^

Additionally, multiple studies have demonstrated that rural communities and critical access hospitals (CAHs) have increased needs with regards to antibiotic usage. Studies have found rural women were 10% more likely to receive an inappropriately long course of antibiotics for UTI infections and 76% of patients seen in CAHs received inappropriate antibiotics for asymptomatic bacteriuria.^4,5^ Overall, antibiotic usage at small community hospitals was similar to large community hospitals with a lower case-mix index, demonstrating overprescription at small community hospitals.^6^

To combat this problem, The Centers for Medicaid and Medicare Services (CMS) instituted a requirement for Antibiotic Stewardship Programs (ASP) at all acute care and CAHs.^7^ Small hospitals often lack robust ASPs, and further face a variety of challenges including a lack of infectious diseases (ID) trained clinicians and pharmacists, geographic isolation, and competing priorities leading to lower buy-in from leadership and senior staff. ^6^

Multiple groups including the CDC and Infectious Disease Society of America have supported the use of remote telehealth-based programs to implement ASP programs in these settings.^2,8^ These remote based ASP interventions have previously been implemented in many settings across the globe, and have consistently demonstrated high levels of provider uptake, reductions in antibiotics prescribed, and reductions in costs related to antibiotic usage.^9–13^ In addition to demonstrating improvements in patient based and cost based indicators, qualitative measurements have previously shown remote ASP programs help build community amongst rural CAH’s and help increase staff education and change antibiotic usage.^14^

## Methods

This study was conducted across CHI Health facilities in Minnesota and North Dakota, encompassing one acute care hospital (280 beds) and thirteen CAHs (25 beds or less). Historically, while the acute care hospital had an ID consultation service available Monday-Friday through locum ID providers, they did not have an ID pharmacist, and the critical access sites lacked any dedicated onsite ID-trained providers or pharmacists. Recognizing that The Joint Commission (TJC) and Centers for Medicare and Medicaid Services (CMS) require antimicrobial stewardship programs (ASPs) for all acute and critical access facilities, responsibility to meet these requirements often fell to an onsite pharmacist in addition to their usual daily requirements. These pharmacists typically had limited dedicated time for antimicrobial stewardship (AMS) related activities and lacked extensive specialized training in ID or AMS to implement robust programs.

To strengthen these vital programs at our facilities, a remote ASP expansion was initiated at the beginning of fiscal year 2024. This included adding 0.1 FTE ID physician for Minnesota sites, 0.2 FTE ID physician for North Dakota sites, and 1.0 FTE AMS pharmacist to serve all regional sites. The AMS pharmacist was responsible for providing daily audit and feedback for patients on antimicrobial therapy. The process of providing multi-facility coverage was streamlined by the existing clinical decision support tool, TheraDoc, which was in place at all facilities. TheraDoc was utilized by the AMS pharmacist to generate a list of alerts each morning to prioritize patients for review. The alerts included positive cultures and tests (blood, CDI, influenza, COVID-19), multidrug-resistant organisms including methicillin-resistant *Staphylococcus aureus* (MRSA), vancomycin-resistant *Enterococcus* (VRE), extended-spectrum beta lactamase (ESBL), and carbapenem-resistant *Enterobacteriaceae* (CRE), targeted/restricted antimicrobials, prolonged therapy (>72 h), bug-drug mismatches, duplicate coverage, IV-to-PO conversions, and de-escalation opportunities. Complex patient cases requiring intervention were discussed with ID physicians during daily stewardship telephone rounds. Following these rounds, the AMS pharmacist would discuss the recommendations with the local pharmacist, and the recommendations would then be relayed to the local provider either by the AMS or onsite pharmacist. Intervention descriptions and acceptance or rejection of the interventions were documented within the electronic medical record. For many patients, the AMS team would continue following their clinical course throughout their hospitalization and provide additional recommendations as needed. In addition to reviewing patients identified through the TheraDoc alerts, the AMS team would review additional patient cases at the request of the onsite providers.

The antimicrobial stewardship program’s effectiveness was evaluated by comparing fiscal year 2023 and 2024 data across key metrics: antimicrobial utilization, quantified as days of therapy (DOT) per 1,000 patient days; antimicrobial expenditures, measured as antibiotic cost per case-mix index (CMI) adjusted patient day; and hospital-acquired CDI rates. χ^2^ tests were utilized to determine the significance of outcomes, and a *P* value < .05 was considered statistically significant. For antimicrobial utilization analysis, we specifically focused on vancomycin, meropenem, linezolid, and aztreonam. The latter three agents, all restricted within our hospital system, were prioritized due to observed instances of use outside of their established restriction criteria. Details of AMS interventions were also tracked, including type of intervention, indication, and acceptance rate.

## Results

**Figure 1.**
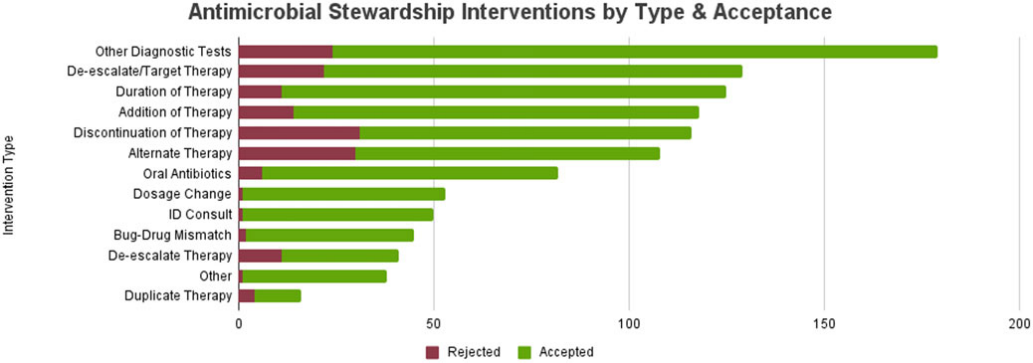
Antimicrobial interventions by type and acceptance in fiscal year 2024 after remote stewardship implementation.

**Figure 2.**
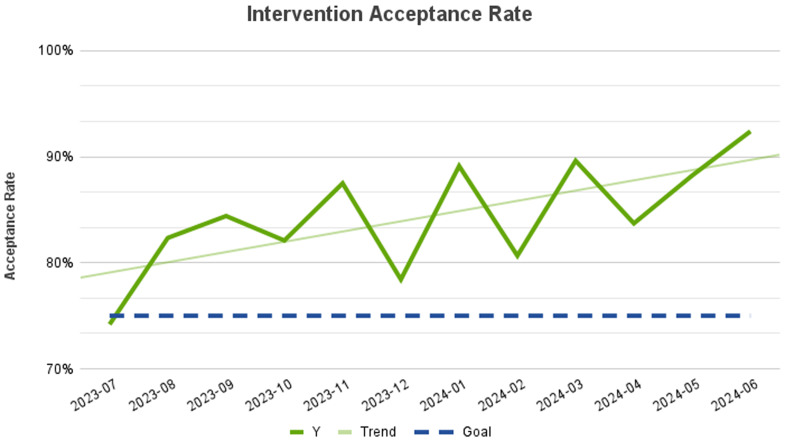
Monthly intervention acceptance rate in fiscal year 2024.

**Figure 3.**
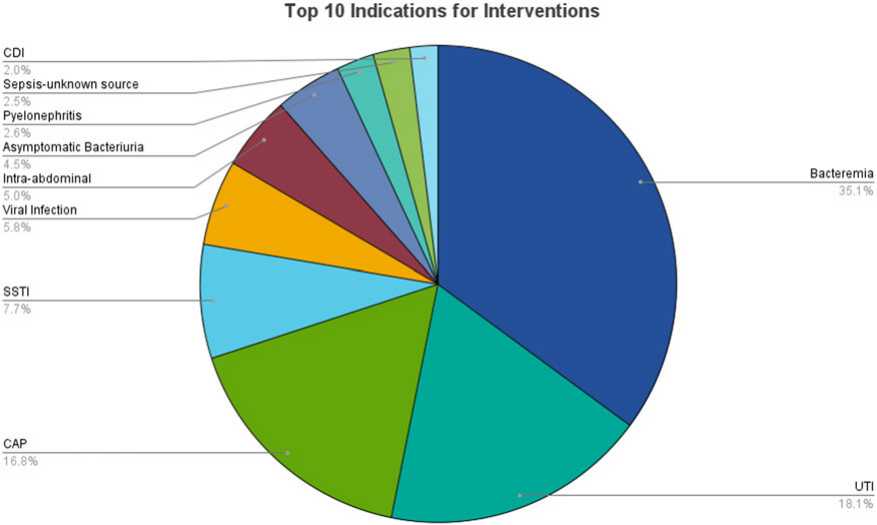
Top 10 indications for stewardship interventions.

In the first twelve months following regional remote stewardship expansion, the program averaged 42 interventions per month in CAHs with an intervention acceptance rate of 83% and 50 interventions per month in the acute care hospital with an acceptance rate of 88%, totaling 92 interventions per month, with an overall intervention acceptance rate of 86%. The most common types of interventions included other diagnostic testing, which included recommendations to obtain cultures, imaging, and other laboratory tests such as viral testing, procalcitonin and urine antigens (179), de-escalate/target therapy based on culture results (129), duration of therapy (125), addition of therapy (118), and discontinuation of therapy (116) (Figure 1). The interventions accepted at the highest rate were dosage changes (98%) and ID consult (98%). The interventions accepted at the lowest rate were discontinuation of therapy (73%) and alternate therapy (72%), which included recommendations for changing to an alternative antibiotic choice that was not necessarily an escalation or de-escalation in therapy, but perhaps a more appropriate choice depending on indication, facility antibiogram, and patient specific factors. The average intervention acceptance rate also increased from the expansion of the remote ASP through the first year (Figure 2). The most common indications for intervention were bacteremia (35.1%), UTI (18.1%), and CAP (16.8%) (Figure 3).

**Figure 4.**
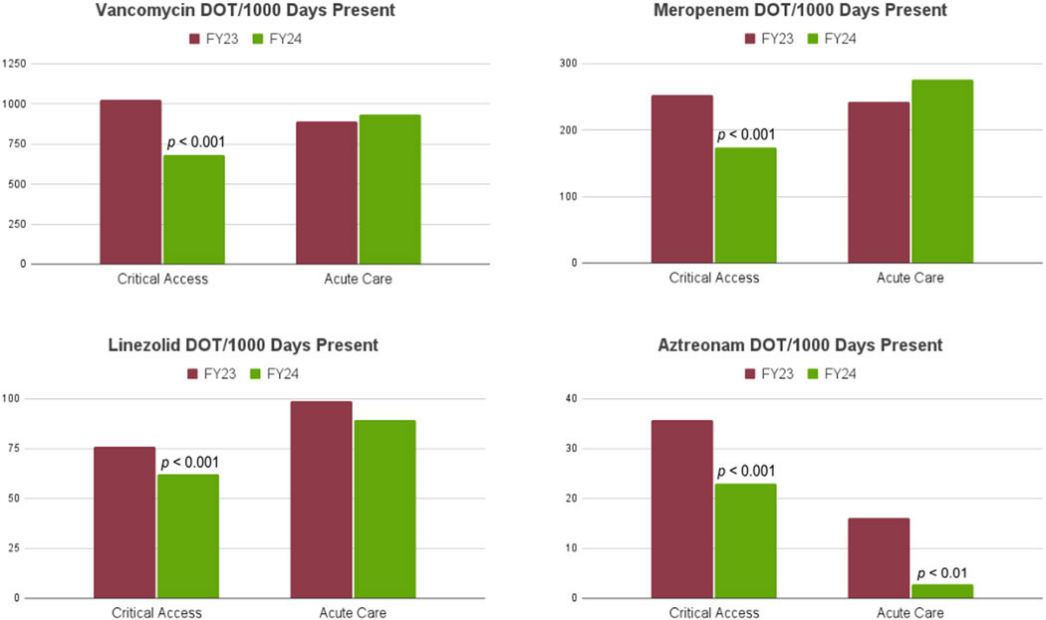
Antimicrobial use (days of therapy/1,000 patient days) for vancomycin, meropenem, linezolid, and aztreonam before (FY23) and after (FY24) remote antimicrobial stewardship implementation.

**Figure 5.**
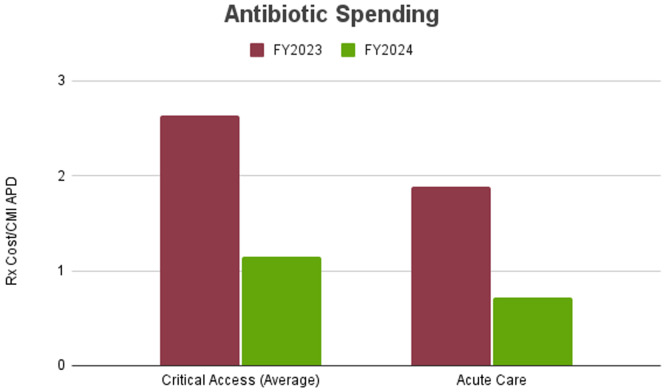
Rx cost/CMI adjusted patient days before (FY2023) and after (FY2024) remote antimicrobial stewardship implementation.

In the acute care facility, only aztreonam use was significantly reduced (*P* < .01) (Figure 4). There were no significant changes in vancomycin, meropenem, or linezolid usage from FY23 to FY24, although we did observe a general trend towards decreased use of linezolid, piperacillin/tazobactam, ceftriaxone, and fluoroquinolones. In the CAHs, there were significant reductions (*P* < .001) in use of vancomycin (33%), meropenem (31%), linezolid (18%), and aztreonam (35%). Among all facilities, there was a 53% reduction in hospital-acquired CDI rates, with 15 cases in FY23 compared to 7 cases in FY24 (*P* < .01) There were also significant reductions in antibiotic cost in both critical access and acute care hospitals (Figure 5). In CAHs, the average antibiotic cost per Case Mix Index Adjusted Patient Days fell from $2.64 to $1.15. In the acute care hospital, it fell from $1.89 to $0.72. In total, the ASP demonstrated a $619,053.97 reduction in antimicrobial expenses.

## Discussion

This study demonstrates the positive impact of implementing a remote antimicrobial stewardship program, particularly for CAHs lacking readily available ID specialists. Program success was evident through reductions in both antimicrobial expenses and the incidence of CDI. Additionally, a substantial improvement in the program’s acceptance rate over the 12-month study period highlights the value of frequent collaboration with onsite colleagues and suggests growing confidence and trust in the remote stewardship service over time. Regarding broad-spectrum antibiotic reduction, there was a greater impact seen at the critical access sites, further demonstrating the high level of need for stewardship programs at these facilities. Furthermore, given that these sites are typically widely distributed and often remote from major urban centers, a remote stewardship program represents an ideal solution to antimicrobial optimization in these settings.

It is also worth highlighting the variety of stewardship interventions made. While de-escalation and discontinuation of antimicrobial therapy were frequent interventions in this study and are traditionally emphasized in stewardship practices, it is particularly noteworthy that “Other Diagnostic Tests” and “Addition of Therapy” were also among the most frequent intervention types, both with high acceptance rates. This highlights how the stewardship team actively identifies gaps in diagnostic workup and instances where current antimicrobial coverage is inadequate or incorrect and recommends additions to optimize patient treatment. Involvement of ID physicians in the ASP was essential for program success in general but was particularly helpful for recommending diagnostics, as their expertise was often sought out by onsite teams for input on additional workup.

There were some limitations to this study. While it may be nice to have information regarding ASP impact on additional patient outcomes, we did not include a comparative analysis of length of stay or mortality data given the numerous confounding variables that influence these metrics beyond simple antimicrobial usage. Critical access facilities typically have shorter lengths of stay, with many patients either discharging promptly or transferring to a higher level care. Additionally, the frequent accommodation of patients receiving extended courses of intravenous antimicrobials in “swing beds” at these sites further complicates the interpretation of average length of stay data in the context of stewardship involvement. While we did not evaluate these additional patient outcomes with this study, they could be further reviewed for impact in future analysis, particularly at our acute care facility where there are potentially less confounders. The study also did not assess for additional intervention measure that could have contributed to the observed reduction in CDI, such as various infection control measures.

In summary, utilizing remote antimicrobial stewardship services is a unique and viable method for expanding stewardship coverage to resource-limited facilities and can positively impact both patient-focused and financial outcomes, particularly at CAHs that do not have ID specialists available onsite.
